# Epithelial-derived factors induce muscularis mucosa of human induced pluripotent stem cell-derived gastric organoids

**DOI:** 10.1016/j.stemcr.2022.02.002

**Published:** 2022-03-03

**Authors:** Keiichiro Uehara, Michiyo Koyanagi-Aoi, Takahiro Koide, Tomoo Itoh, Takashi Aoi

**Affiliations:** 1 Division of Advanced Medical Science, Graduate School of Science, Technology and Innovation, Kobe University, Kobe 6500017, Japan; 2 Department of iPS Cell Applications, Graduate School of Medicine, Kobe University, Kobe 6500017, Japan; 3 Department of Diagnostic Pathology, Graduate School of Medicine, Kobe University, Kobe 6500017, Japan; 4 Center for Human Resource Development for Regenerative Medicine, Kobe University Hospital, Kobe 6500017, Japan; 5 Division of Gastrointestinal Surgery, Department of Surgery, Graduate School of Medicine, Kobe University, Kobe 6500017, Japan

**Keywords:** hiPSC, gastric organoid, muscularis mucosa, Hedgehog signal, TGF-β, signal, mechanical property

## Abstract

Human gastric development has not been well studied. The generation of human pluripotent stem cell-derived gastric organoids (hGOs) comprising gastric marker-expressing epithelium without an apparent smooth muscle (SM) structure has been reported. We modified previously reported protocols to generate hGOs with muscularis mucosa (MM) from hiPSCs. Time course analyses revealed that epithelium development occurred prior to MM formation. Sonic hedgehog (SHH) and TGF-β1 were secreted by the epithelium. HH and TGF-β signal inhibition prevented subepithelial MM formation. A mechanical property of the substrate promoted SM differentiation around hGOs in the presence of TGF-β. TGF-β signaling was shown to influence the HH signaling and mechanical properties. In addition, clinical specimen findings suggested the involvement of TGF-β signaling in MM formation in recovering gastric ulcers. HH and TGF-β signaling from the epithelium to the stroma and the mechanical properties of the subepithelial environment may influence the emergence of MM in human stomach tissue.

## Highlights

• We successfully induce hGOs with MM by culturing for 8 weeks
• The emergence of MM occurs after epithelial maturation
• HH, TGF-β, and the mechanical environment are suggested to be involved in MM formation

## Graphical abstract

**Figure:**
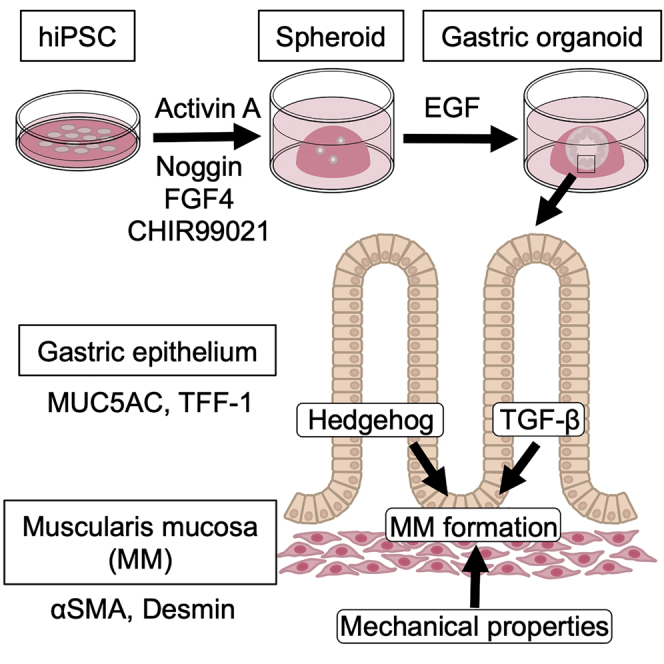

## Teaser

In this article, Aoi and colleagues successfully induce hiPSC-derived human gastric organoids with muscularis mucosa (MM), which allow us to investigate human gastric MM development. A time course analysis revealed that MM differentiates after epithelial maturation. Hedgehog and TGF-β signaling from the epithelium as well as the mechanical environment may be involved in the MM formation of human stomach tissue

## Introduction

The gastric muscularis mucosa (MM), which is contained within the mucosa, is a thin layer of smooth muscle (SM) that separates the mucosa from the submucosa (Ross and Pawlina, 2020). The function of the gastric MM is to help expel the contents of the deeper gastric glands, prevent clogging, and enhance contact between the gastric epithelium and the contents of the gastric lumen for absorption (Young et al., 2014). In gastric cancer, the rates of lymph node metastasis are higher in submucosal cases than in intramucosal cases; therefore, in cancer staging, the T category is changed if gastric cancers invade beyond the MM (Gotoda et al., 2000). In gastric ulcer, if the damage is confined to the mucosa, gastric mucosa can be completely restored without scarring. However, damage that reaches a deeper part of the stomach wall beyond the MM can cause fibrosis, and a depressed pit can be formed (Rosai et al., 2011). Thus, the gastric MM plays important roles in both normal and pathological states.

Several signaling pathways regulating endoderm patterning, gastric specification, stomach regionalization, and morphogenesis have been reported to be involved in gastric development (McCracken and Wells, 2017); however, the details are not fully understood, especially concerning the gastrointestinal mesenchymal development. There are some reports on the formation of the muscularis propria in model animals that have demonstrated the involvement of Hedgehog (HH), Transforming growth factor beta (TGF-β), and Barx1 in mesenchymal and SM differentiation of the gut in chicks, mice, and zebrafish (Aubin et al., 2002; Fukuda et al., 1998; Gays et al., 2017; Jayewickreme and Shivdasani, 2015; Mao et al., 2010; Ramalho-Santos et al., 2000). However, how the human stomach develops in the embryo stage and how the human gastric MM is induced remain to be clarified.

The scarcity of specimens of human embryo and fetal organs has hindered studies on human developmental biology. In previous decades, various organs and tissues derived from the endoderm, mesoderm, and ectoderm have become able to be induced from human pluripotent stem cells, such as human embryonic stem cells (hESCs) and human induced pluripotent stem cells (hiPSCs) (Koterazawa et al., 2020; Shi et al., 2017; Suzuki et al., 2019), which has allowed us to investigate normal and pathological human developmental processes and their underlying mechanisms. McCracken et al. reported the generation of human pluripotent stem cell-derived gastric organoids (hGOs); however, the hGOs lacked subepithelial SM tissue (McCracken et al., 2014).

In the present study, we successfully generated GOs with MM from hiPSCs by culturing for a longer duration than in previous reports. Furthermore, using this induction method, we revealed that epithelial-derived HH signaling, TGF-β signaling, and the mechanical properties of the substrate worked together to induce MM formation in hiPSC-derived hGOs.

## Results

### Induction of hGOs accompanied by the subepithelial SM layer

**Figure 1:**
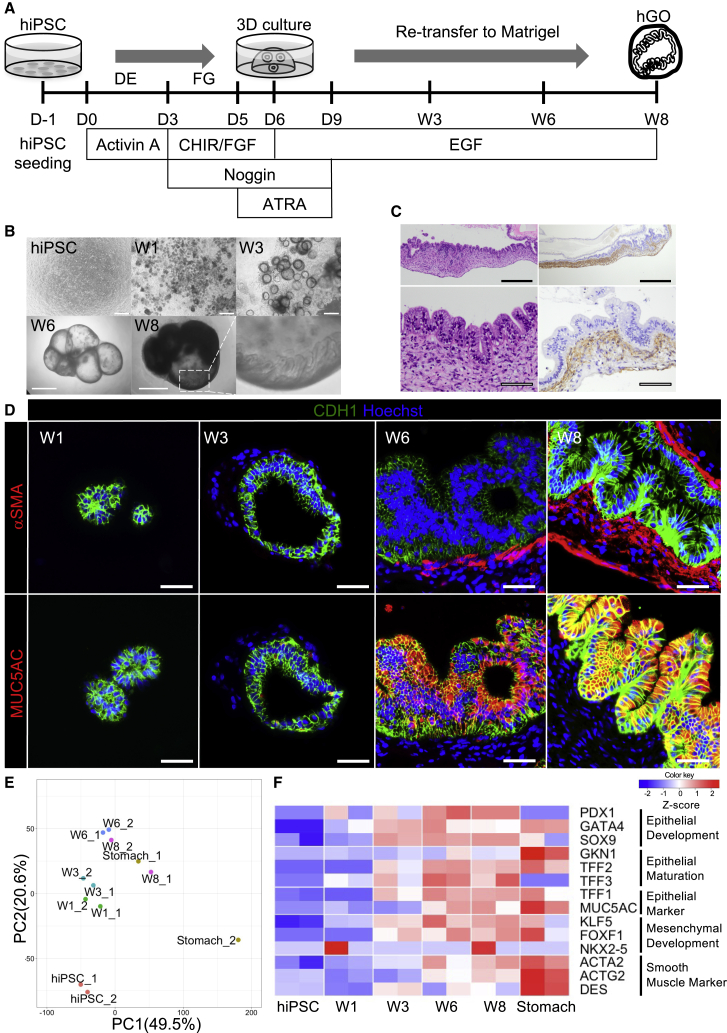
Induction of human gastric organoids (hGOs) with muscularis mucosa (MM) from human induced pluripotent stem cells (hiPSCs) (A) A schematic diagram of hGOs with MM generated from hiPSCs using three-dimensional culture with Matrigel. (B) Morphological changes during the differentiation of hGOs. Stereomicrographs showed that the epithelium of hGOs had a folded and glandular architecture at week 8. Scale bars, 500 μm (hiPSCs; weeks 1 and 3) and 2 mm (weeks 6 and 8). (C) Hematoxylin and eosin (H&E) staining and αSMA immunohistochemical (IHC) staining of hGO with MM at week 8 revealed folded epithelium that resembled gastric foveolae or glands. Subepithelial spindle cells were arranged like a bundle on αSMA IHC staining. Scale bars, 500 μm (upper panel) and 50 μm (lower panel). (D) Immunohistochemistry (IHC) of hGOs. After week 3, hGOs were accompanied by subepithelial αSMA-positive spindle cells (MM), and the epithelium of hGOs was positive for MUC5AC. Scale bars, 50 μm. (E) A principal component analysis of the RNA-seq data of hiPSCs, differentiated derivatives into stomach organoids (weeks 1, 3, 6, and 8) that were obtained in two independent differentiated experiments, and two stomach samples. The horizontal axis represents PC1 scores, while the vertical axis represents PC2 scores. (F) Heatmap showing changes in the expression of gastric epithelial development, maturation, mesenchymal development, SM, and gastric epithelial markers of RNA-seq data.

First, we performed differentiation of hiPSCs into gastric lineage according to previous reports (McCracken et al., 2011, 2014, 2017). In brief, hiPSCs were differentiated into definitive endoderm (DE) for the first three days using Activin A. Subsequently, DE was differentiated to posterior foregut endoderm (FG) by inhibitor of glycogen synthase kinase 3 (CHIR99021), FGF4, and Noggin. Posterior foregut spheroids were transferred to a Matrigel-based three-dimensional (3D) culture. We then maintained the 3D culture for 7 weeks, which was 3 weeks longer than in the previously reported study (McCracken et al., 2014) (Figure 1A).

On stereomicroscope observation, the epithelium of the derivatives of hiPSCs showed increasingly complex and folded structures from 6 to 8 weeks (Figure 1B). Histological examinations by hematoxylin and eosin (H&E) staining at week 8 also revealed that the epithelium formed glandular or foveolar structures (Figure 1C), as in the previous report (McCracken et al., 2014). From weeks 6 to 8, the epithelium of the differentiated structures became positive for the gastric epithelial markers MUC5AC (Figure 1D, lower panel) and TFF-1 (Rio et al., 1988) (Figure S1A, lower panel). Notably, subepithelial αSMA-positive spindle cells began to appear at week 6 and formed a band-like structure that resembled MM at week 8 (Figures 1C and 1D, upper panel). Subepithelial spindle cells were found to be positive for Desmin, another muscle marker, at week 8 (Figure S1A, upper panel). Some of the subepithelial spindle cells were positive for histamine receptor H1 (HRH1), which mediates the contraction of SMs (Figure S1B). Based on these results, we considered the structure to correspond to gastric MM and thus successfully generated hGOs with MM from hiPSCs.

To clarify the gene expression dynamics during differentiation into hGO with MM, an RNA-seq analysis was performed at different time-lapse sampling points (hiPSC, week 1, week 3, week 6, and week 8). According to a principal component analysis (PCA), which enables the visual assessment of the similarities and differences between samples to determine whether or not samples can be grouped (Ringnér, 2008), we noted that the stomach samples showed variability, and the hGOs with MM at week 8 were similar to one of the two stomach samples (Figure 1E), with hiPSCs gradually differentiated toward the stomach. Consistent with the immunohistochemical findings that subepithelial αSMA-positive cells appeared following CDH1-positive epithelial cells (Figure 1D), the heatmap showed that gastric epithelial and mesenchymal development, maturation markers, and SM markers were gradually increased up to week 8, and their expression became similar to that in the stomach. The expression of *TFF-1*, a gastric surface mucous cell marker (Aihara et al., 2017), preceded the expression of SM markers after week 3 (Figure 1F). These findings indicated that subepithelial SM cells begin to differentiate after gastric epithelial maturation, and some epithelial-derived factors might have been involved in the differentiation of subepithelial SM cells (MM) after week 3.

Human GOs with MM enabled us to examine the factors involved in MM differentiation, and we focused on three candidates: HH signaling, TGF-β signaling, and the mechanical properties of the substrate, according to previous reports on SM differentiation.

### Cyclopamine inhibited MM formation of hGOs

**Figure 2:**
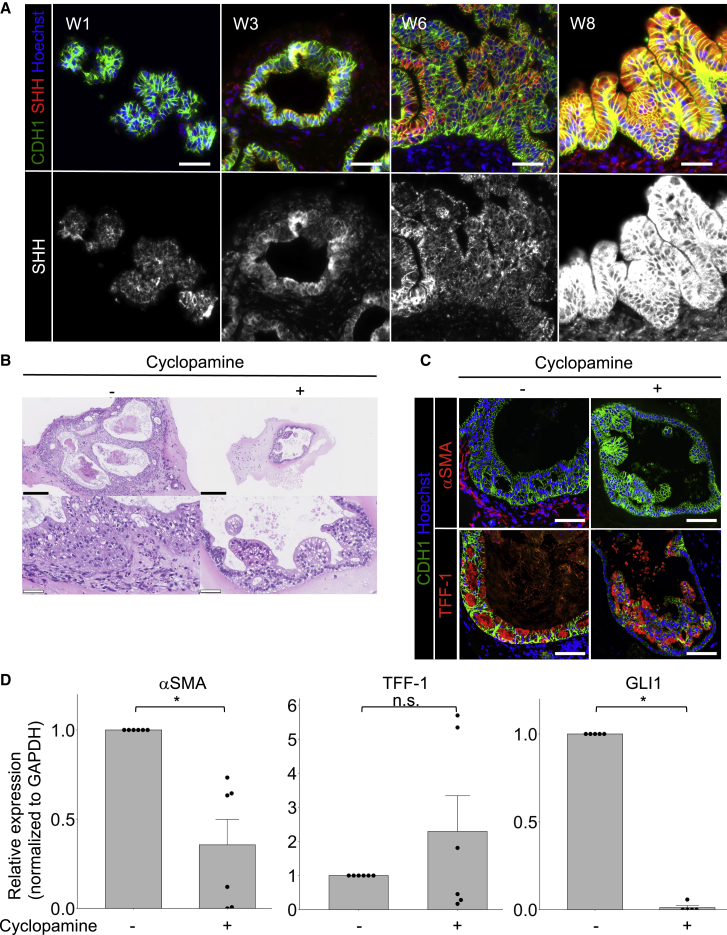
Muscularis mucosa formation of human gastric organoids (hGOs) was reduced by cyclopamine (Hedgehog signal inhibitor) (A) Immunohistochemistry of Sonic hedgehog (SHH) during induction of hGOs with MM. After week 3, the SHH expression was found mainly in the organoid epithelium, and IHC positivity for SHH in epithelial cells gradually increased toward week 8. Scale bars, 100 μm. (B) H&E staining of hGOs with and without cyclopamine treatment. The hGOs treated with cyclopamine were smaller and showed fewer subepithelial cells than those without cyclopamine treatment. Scale bars 250 μm (upper panels) and 50 μm (lower panels). (C) IHC of hGOs with and without cyclopamine treatment. In hGOs with cyclopamine treatment, subepithelial αSMA-positive cells were not found, and MM formation was reduced. TFF-1 expression was found in the epithelium of both hGOs, and gastric differentiation was preserved. Scale bars, 100 μm. (D) qRT-PCR of hGOs with and without cyclopamine treatment. The expression of αSMA (MM marker) and GLI1 (downstream molecule of HH signaling) was reduced by cyclopamine. TFF-1 (gastric epithelial marker) was not significantly changed. αSMA, number of independent differentiation experiments: n = 6; GLI1, n = 5; TFF-1, n = 6. Mean values ± SE, unpaired t test: ^∗^p < 0.01.

To examine whether or not the HH signaling, which has been reported to be involved in gut mesenchymal SM formation of chicks and mice (Kolterud et al., 2009; Mao et al., 2010; Ramalho-Santos et al., 2000), was involved in MM formation, we assessed the changes in the Sonic hedgehog (SHH) expression during the induction of hGO with MM by immunohistochemistry (IHC) and *in situ* hybridization (ISH). We first confirmed that IHC appropriately worked using negative and positive controls (Figure S2). As shown in Figure 2A, positivity of IHC for SHH gradually increased from weeks 3 to 8 and were found in the epithelium, indicated by CDH1-positivity. Furthermore, ISH revealed *SHH* mRNA in the epithelium at week 6 (Figures S3A and S3B). These data suggested that SHH was mainly synthesized in and secreted by the epithelium. We also performed immunostaining of Indian hedgehog (IHH), as shown in Figure S3C. As with SHH, IHH expression was mainly observed in the epithelium at week 3, and the IHC positivity for IHH increased in the epithelial cells at weeks 6 and 8.

To examine the role of HH during the induction of hGOs with MM, we added cyclopamine, an HH signaling inhibitor, to the medium from week 3. Although the gastric foveolar-like architecture was preserved under the administration of cyclopamine, hGOs treated with cyclopamine tended to be smaller, and there were fewer subepithelial cells constituting the hGOs in comparison with those that were not treated with cyclopamine, according to a histological examination (Figure 2B).

Immunohistochemistry revealed that αSMA-positive subepithelial spindle cells were decreased by inhibiting HH signaling. An MM-like structure, with an αSMA-positive spindle cell bundle, was not found in hGOs treated with cyclopamine (Figure 2C upper panels). Hoechst staining also showed that cyclopamine treatment resulted in the defect of the subepithelial cells. Epithelial TFF-1-positive cells were observed regardless of cyclopamine, indicating that gastric epithelial differentiation was preserved even with HH signaling inhibition (Figure 2C lower panels). A quantitative RT (qRT)-PCR analysis also showed that cyclopamine reduced the *αSMA* expression, whereas the expression of *TFF-1* and *TFF-2*, another gastric epithelial marker (Uhlen et al., 2015), was not significantly changed (Figures 2D and S3D). The expression of *GLI1*, a known downstream molecule of HH signaling, was significantly decreased, indicating that HH signaling was successfully inhibited by cyclopamine (Figure 2D).

In addition, the expression of *Myocardin* (*MYOCD*), which is a master transcriptional regulator of SM differentiation and has been reported to be a direct target of HH signaling in intestinal mesenchymal cells (Cotton et al., 2017; Wang et al., 2003; Zacharias et al., 2011), was also reduced in the hGOs by cyclopamine (Figure S3E). These results suggested that HH secreted from the epithelium may act on the subepithelial cells to activate HH signaling and increase the expression of αSMA via MYOCD.

### SB431542, an inhibitor of TGF-βR1, reduced MM of hGOs

**Figure 3:**
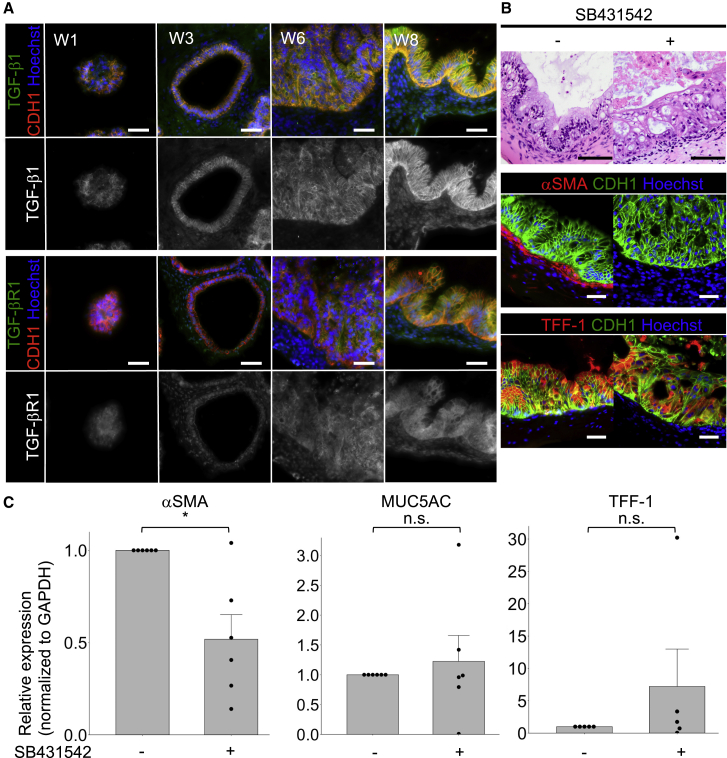
MM differentiation of hGOs was reduced by SB431542 (TGF-βR1 inhibitor) (A) IHC of TGF-β1 and TGF-βR1 of hGOs with MM during induction. The TGF-β1 expression was mainly found in the epithelia of hGOs. TGF-βR1 was weakly positive in not only the epithelia but also subepithelial cells. Scale bars, 50 μm. (B) H&E staining and IHC of αSMA and TFF-1 of hGOs with the treatment of SB431542. αSMA expression was not found in subepithelial cells, and MM differentiation was inhibited by SB431542. TFF-1 expression was positive in the epithelia, and gastric differentiation was preserved under SB431542. Scale bars, 50 μm. (C) Results of a qRT-PCR analysis of αSMA, MUC5AC, and TFF-1 of hGOs treated with SB431542. αSMA expression was reduced by SB431542, whereas the MUC5AC and TFF-1 expression was not significantly changed. αSMA, number of independent differentiation experiments: n = 6; MUC5AC, n = 6; TFF-1, n = 5. Mean values ± SE, unpaired t test: ^∗^p < 0.01.

We next evaluated whether or not TGF-β signaling was involved in MM formation. Although TGF-β signaling has been reported to be involved in gastrointestinal SM differentiation in zebrafish and vascular SM differentiation in humans (Gays et al., 2017; Guo and Chen, 2012), the relationship between TGF-β signaling and human gastric mesenchymal differentiation has not been clarified. To assess the expression of TGF-β1 and TGF-βR1 during gastric differentiation in our hGO development, immunohistochemistry was performed. Regarding TGF-β1 IHC findings, we confirmed that IHC appropriately detected TGF-β1 using negative and positive controls (Figure S4). TGF-β1 was largely detected in the epithelium of hGOs, and IHC positivity for TGF-β1 in the epithelium increased from weeks 3 to 8 (Figure 3A upper panels). Furthermore, ISH at weeks 6 and 8 revealed that *TGF-β1* was synthesized *de novo* predominantly in the epithelium rather than in subepithelial cells (Figures S5A and S5B). The IHC positivity for TGF-βR1 in hGOs was also gradually increasing, and subepithelial cells were weakly positive after week 3 (Figure 3A lower panels).

We next administered SB431542, an inhibitor of TGF-βR1, from week 3 and examined the induced hGOs at week 8, since the MM of hGOs appeared after week 3. Immunohistochemical staining revealed that the epithelium of the hGOs was positive for the gastric epithelial marker TFF-1, and gastric differentiation was conserved under the addition of SB431542. However, αSMA-positive subepithelial cells were not detected (Figure 3B), indicating that the MM formation of hGOs was suppressed by inhibition of TGF-β signaling. A qRT-PCR analysis showed that the expression of *αSMA* was significantly decreased by the addition of SB431542, whereas the gastric epithelial differentiation was preserved, as the *MUC5AC* and *TFF-1* expression did not significantly change (Figure 3C). The expression of αSMA was also reduced by SB431542 at the protein level (Figure S6).

These data suggest that SB431542 reduced the MM induction of hGOs after week 3, and epithelial-derived TGF-β signaling might be involved in human gastric MM differentiation.

### Mechanical environment promotes subepithelial αSMA expression

**Figure 4:**
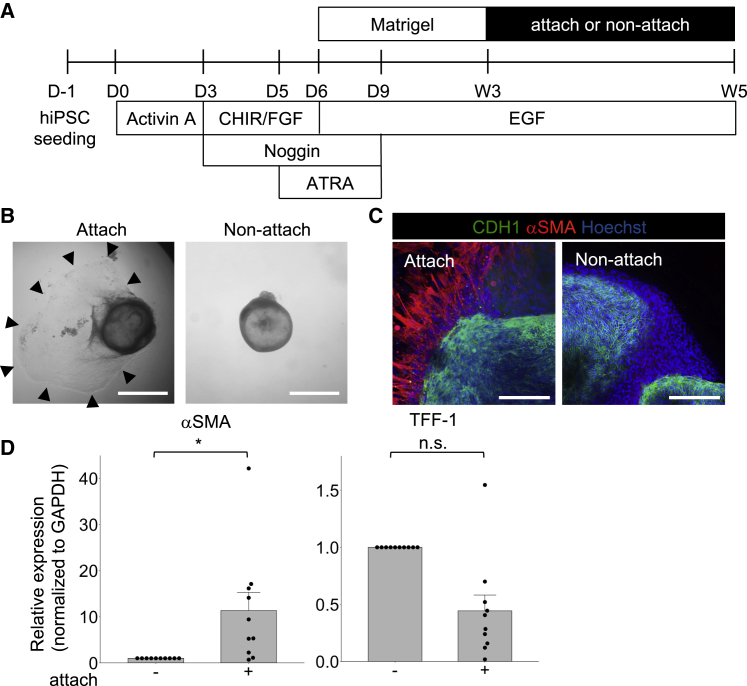
Attaching to the surface of the dishes affects MM differentiation of hGOs (A) A schematic diagram of the generated hGOs to investigate the effect of attaching to the surface of the dishes. (B) Stereomicrographs showed spreading cells around the hGO with attaching to the surface of the dishes. Scale bars, 1 mm. (C) Immunohistochemical staining showed that the spreading cells were positive for αSMA in the hGOs with attachment. αSMA-positive cells were not found around the hGOs without attachment. Scale bars, 200 μm. (D) A qRT-PCR analysis of hGOs with and without attachment. Attaching (substrate property) promoted αSMA expression. The TFF-1 expression was preserved. n = 10 independent experiments, mean values ± SE, unpaired t test: ^∗^p < 0.001

During their development, our hGOs were maintained carefully within the Matrigel in order to prevent contact with the surface of the dishes. However, they occasionally unintentionally penetrated the Matrigel and contacted the surface of the dishes. On such occasions, we noticed that spindle cells spread around the hGOs (data not shown), prompting the hypothesis that attachment to the surface of dishes induces SM differentiation. To test this hypothesis, we transferred the hGOs that developed in the Matrigel at week 3 to an ultra-low-attachment plate or attachment plate without re-embedding in the Matrigel and maintained them for 2 weeks (Figure 4A) before examining the SM differentiation by immunohistochemistry, qRT-PCR, and western blotting.

While the hGOs on the ultra-low-attachment plate were not surrounded by spreading cells, the hGOs on the attachment plates were surrounded by proliferating spindle cells right after the transfer (Figure 4B). As we hypothesized, immunohistochemistry revealed that only the spindle cells around the hGOs that had attached to the surface of the dishes were positive for αSMA (Figure 4C). Consistent with immunohistochemistry, western blotting and qRT-PCR showed that attachment of the hGOs to the surface of the dishes resulted in the upregulated expression of αSMA (Figures 4D and S7A).

The next issue to be addressed was which cells in hGOs were affected by the mechanical environment. Mesenchymal stem cells (MSCs) on stiff substrate reportedly differentiate into SM cells in response to TGF-β (Park et al., 2011). Our hGOs at week 3 had some subepithelial cells that were positive for the MSC markers CD146 or CD44 (Crisan et al., 2008) (Figure S7B), suggesting that the subepithelial MSCs in the hGOs were affected by the mechanical environment under the epithelial-derived TGF-β and thereby differentiated into SM cells.

### Effects of TGF-β signaling on HH signaling and mechanical properties

We next examined the relationship between HH and TGF-β signaling in hGOs using the TGF-β inhibitor SB431542. TGF-β reportedly induces the expression of the HH signaling molecule GLI2 in various human cell types and cancer cell lines (Dennler et al., 2007). In addition, GLI1 is a downstream target of HH signaling.

**Figure 5:**
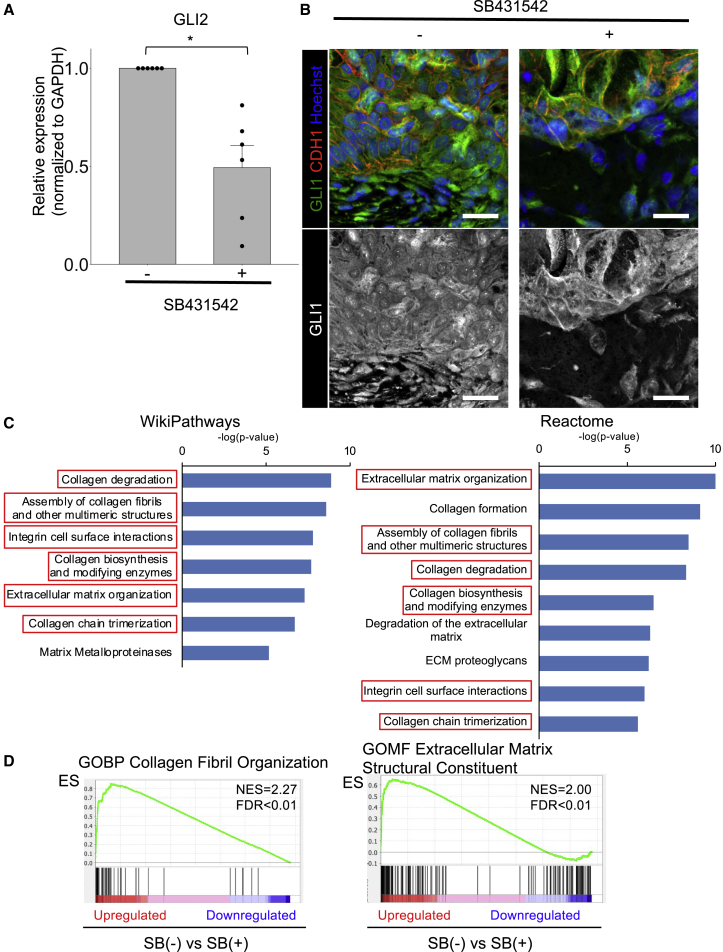
Effects of TGF-β signaling on HH signaling and mechanical properties (A) A qRT-PCR analysis of GLI2 in hGOs with or without the treatment of SB431542. The GLI2 expression was reduced by SB431542. Normalized to control. n = 6 independent experiments, mean values ± SE, unpaired t test, ^∗^p < 0.01. (B) According to IHC of hGOs, the subepithelial GLI1 expression was reduced compared with hGOs without SB431542 treatment. Scale bars, 20 μm. (C) A pathway analysis (WikiPathways and Reactome) for genes of RNA-seq data downregulated more than 10-fold by treatment of SB431542. Pathways with -log10 p value >5 were listed. (D) A gene set enrichment analysis (GSEA) of RNA-seq data showed that gene sets associated with collagen fibril organization (in the biological process [BP]) and extracellular matrix structure constituent (in the molecular function [MF]) were negatively regulated by SB431542.

The *GLI2* mRNA expression was significantly reduced by SB431542 treatment according to qRT-PCR of six independent experiments (Figure 5A), indicating the successful suppression of TGF-β signaling in the hGOs. Although the *GLI1* mRNA expression was not significantly changed (data not shown), GLI1-positive subepithelial spindle cells of hGOs treated with SB431542 were reduced compared with the subepithelial cells of hGOs without SB431542 in immunohistochemistry (Figure 5B). These results indicated that TGF-β and HH signaling act not only independently but also collaboratively in the induction of MM in hGOs.

To broadly investigate the effects of TGF-β signaling inhibition on hGO induction, we analyzed the global gene expression changes in hGOs after the treatment of SB431542 by RNA-seq. We confirmed the reduced expression of GLI2 (data not shown) and identified 70 upregulated genes and 66 downregulated genes (fold change >10) by the treatment of SB431542. For these differentially expressed genes, we performed a pathway enrichment analysis on WikiPathways and Reactome, and pathways with a p value <10^−5^ were defined as significantly affected (listed in Figure 5C). As a result, six categories (collagen degradation, assembly of collagen fibrils and other multimeric structures, integrin cell surface interactions, collagen biosynthesis and modifying enzymes, extracellular matrix organization, and collagen trimerization) were commonly selected as associated with the downregulated genes. In addition, a gene set enrichment analysis (GSEA) showed that gene sets concerning “Collagen Fibril Organization” and “Extracellular Matrix Structural Constituent” were regulated negatively by SB431542 (Figure 5D). These results suggested that TGF-β signaling may also contribute to extracellular matrix stiffness and thus promote MM of hGOs.

### Regeneration of MM in the adult gastric tissues

**Figure 6:**
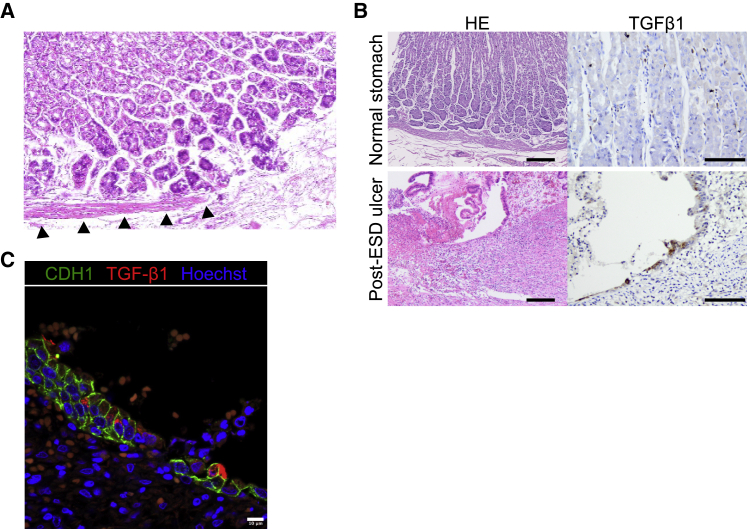
Regeneration of MM in the adult gastric tissues (A) H&E staining of the post-ESD stomach. The black triangles indicate regenerating MM. (B) Representative images of H&E staining and TGF-β1 IHC in normal stomach mucosa and post-ESD ulcer. Scale bars, 200 μm (H&E) and 100 μm (TGF-β1 IHC). (C) Co-immunofluorescence of post-ESD ulcer for CDH1 and TGF-β1. Scale bar, 10 μm.

We demonstrated that epithelial maturation preceded MM induction in our hGOs, which mimicked the gastric development. To investigate whether or not this precedence was found in the regeneration process of adult gastric mucosa, we histologically examined post-endoscopic submucosal dissection (ESD) gastric mucosa. Although gastric epithelium completely regenerates after gastric mucosal injury, gastric MM has not been restored yet (Figure 6A). Consistent with the epithelial development in our organoid model, epithelial regeneration precedes MM, and epithelial-derived factors that induce MM development may also be involved in regeneration.

We demonstrated that TGF-β signaling was involved in gastric MM induction. Generally, some factors that function during development are used for regeneration or disease, and the TGF-β1 expression has been reported to be increased in rats' gastric ulcers (Tominaga et al., 1997). We therefore investigated whether or not TGF-β1 was involved in regeneration of MM. We performed TGF-β1 immunohistochemical staining on post-ESD ulcer specimens. As a control, we also performed immunohistochemistry on a tissue array of normal stomach mucosa that was commercially available. In the histological examination, gastric ulcers or ulcer scars were found in the specimens obtained from post-ESD gastrectomy. When evaluating TGF-β immunohistochemistry, the cases with ≥1% epithelial cells positive for TGF-β1 were considered immunohistochemically positive. Although some inflammatory cells are positive for TGF-β1, only epithelial positivity was evaluated (Figures 6B and 6C) (Li et al., 2007; Tominaga et al., 1997). Four of 24 cases showed positivity in the normal gastric mucosa, and 13 of 18 cases showed positivity in the post-ESD ulcers or ulcer scars (Table 1). The TGF-β1 expression in regenerative epithelium of ulcers or ulcer scars was significantly increased compared with that in the normal mucosa, indicating that epithelial TGF-β1 is involved in not only induction of the developmental process but also regeneration of gastric MM after adult tissue injury.

**Table 1:** Epithelial positivity of TGF-β1 IHC in normal stomach mucosa and post-ESD ulcer or ulcer scar. Fisher exact test

	TGF-β1		p value
	+	–	
Normal stomach	4 (16.7%)	20 (83.3%)	0.00043
Post-ESD ulcer	13 (72.2%)	5 (27.8%)	

## Discussion

In this study, we successfully generated for the first time to our knowledge hiPSC-derived GOs accompanied by MM. We defined the subepithelial bundle consisting of αSMA-positive spindle cells as MM in our hGOs. The human gastric lamina propria contains scattered SM cells and is continuous with the MM (Ross and Pawlina, 2020). In addition, subepithelial myofibroblasts also express SM markers with variable intensity (Powell et al., 2011; Wu et al., 1999). It is thus difficult to clearly distinguish MM from the scattered SM cells and myofibroblasts by the cell marker expression alone without observing the histological structure. To investigate MM formation, immunohistochemical confirmation of the band-like structure of SM cells is essential.

Human PSC-derived GOs reported by McCracken et al. (2014) contained only scattered αSMA-positive cells and no αSMA-positive band-like structure, indicating that MM had not been formed in these GOs. In contrast, the mouse embryonic stem cell-derived GOs reported by Noguchi et al. (2015) and human PSC-derived intestinal organoids reported by Uchida et al. (2017) did show obvious SM marker-positive band-like structures. It took longer to generate GOs consisting of epithelium with MM than those without MM, suggesting that the MM develops later than the epithelium. Our present data indeed demonstrated that the epithelium preceded MM in the organoid development. These findings support the notion that epithelial-derived factors play important roles in MM formation.

We demonstrated in the present study that epithelial-derived TGF-β signaling was involved in SM differentiation of subepithelial cells of hGOs. To our knowledge, TGF-β1 has not been reported to be secreted from gastric epithelium during development or involved in MM induction. However, our analysis of publicly available RNA-seq data (Szabo et al., 2015) showed that *TGF-β1* was expressed in human fetal stomach from 10 to 20 weeks' gestation (data not shown). Indeed, combined with substrate stiffness, TGF-β1 induces mouse mesenchymal cells to differentiate to SM cells through the phosphatidylinositol 3-kinase/Akt pathway and also induces MSCs to differentiate into SM cells (Lien et al., 2006; Park et al., 2011). The subepithelial mesenchymal cells in our hGOs that were positive for mesenchymal stem cell markers might have been induced to differentiate into SM cells by TGF-β and the substrate stiffness.

We showed in the present study that TGF-β signaling inhibition resulted in a significantly suppressed expression of GLI2 but not GLI1. The relationship between HH and TGF-β signaling was previously indicated in mice (Aubin et al., 2002) as well as in cancer and non-cancer cell lines (Dennler et al., 2007). GLI2 was induced rapidly by TGF-β through SMAD in cooperation with β-catenin, and GLI1 was subsequently expressed (Dennler et al., 2009), suggesting that GLI2 is a direct downstream target of TGF-β signaling, while GLI1 is indirectly regulated by TGF-β signaling via HH signaling activation. Although the relationship between TGF-β signaling and HH signaling may partially contribute to subepithelial SM differentiation in hGOs, with various kinds of cells interacting with each other via various signaling pathways, the effect of TGF-β signaling on GLI1 activation in hGOs may be smaller than that in the culture of single cell lines.

The TGF-β1 expression in regenerating human epithelium of post-ESD ulcer was shown in this study. The expression of TGF-β1 as well as collagen, fibronectin, and laminin, was reportedly increased during gastric ulcer healing in rats (Tominaga et al., 1997). These increased extracellular matrix proteins might contribute to the αSMA expression (Tominaga et al., 1997). In mouse gastric ulcers, gastric epithelial HH signaling has also been investigated and shown to be involved in the differentiation of gastric progenitor cells (Kang et al., 2009). However, the role of HH signaling in non-epithelial tissue regeneration in gastric ulcers has not been studied. In the present study, *SHH* decreased from week 6 to 8 in the ISH analyses (Figure S3A), and the inhibition of HH signaling by cyclopamine resulted in the defect of the subepithelial cells (Figures 2B and 2C). Given these findings, HH signaling may be involved in the maintenance of progenitor cells of the subepithelium, but not in the induction of αSMA-positive cells from subepithelial cells. Epithelial-derived factors and their downstream biological processes that play important roles in gastric MM development may help restore the damaged MM in the adult stomach.

In conclusion, HH and TGF-β signaling from epithelium to stroma and the mechanical properties of the subepithelial environment were suggested to be involved in the emergence of MM of human stomach tissue. Furthermore, TGF-β signaling may function during not only the development but also regeneration of MM. In addition, mechanical strain reportedly organizes the SM layer (Huycke et al., 2019). H&E staining and IHC analyses of the αSMA in our hGOs did not appear to demonstrate the formation of the subepithelial layered structure constituted by the lamina propria and MM with a distinct boundary, as seen in human stomach tissues (Figure 1C). The contraction was unclear in our hGOs, probably due to the absence of the muscularis propria. A lack of contraction may result in the incomplete formation of the MM layer by αSMA-positive muscular cells. Further studies may reveal the relationship between these factors and defects in MM reformation in gastric cancer with submucosal invasion.

## Experimental procedures

### hiPSC culture

Two hiPSC lines (201B7 and FF-PB-3AB4) were used in the experiments in this study. The validated hiPSC line 201B7 was purchased from Riken Cell Bank (Tsukuba, Japan) and transferred from on-feeder to feeder-free conditions in our laboratory. The hiPSC line FF-PB-3AB4 was established in our laboratory (Suzuki et al., 2019). These hiPSC lines were cultured according to a previously described method (Nakagawa et al., 2015) with slight modifications. In brief, the hiPSCs were maintained in StemFit AK02N medium (Ajinomoto, Tokyo, Japan) with penicillin (50 units/mL) and streptomycin (50 μg/mL) (#1514022; Gibco, Carlsbad, CA, USA) at 37°C with 5% CO_2_. The medium was changed every other day and passaged every 7 days using 0.5x TrypLE Select (1x TrypLE Select [#A12859-01; Gibco] diluted 1:1 with 0.5 mM EDTA [#06894-14; Nacalai Tesque, Kyoto, Japan]/PBS [-]) and Rho-associated kinase (Rock) inhibitor (Y-27632; WAKO, Osaka, Japan). iMatrix-511 silk (#892021; Nippi, Tokyo, Japan) was used for precoating.

### Gastric differentiation from hiPSCs

Differentiation into the hGOs derived from hiPSCs was performed according to previous reports (McCracken et al., 2014, 2017). In brief, 2.0 × 10^5^ hiPSCs were plated as single cells in a 24-well dish (#142475; Thermo Scientific, Waltham, MA, USA) precoated with iMatrix-511 silk (0.5 μg/cm^2^) in StemFit medium with 10 μM of ROCK inhibitor Y-27632. To induce differentiation into DE, the medium was changed to RPMI-1640 medium (#30264-56; Nacalai Tesque) supplemented with Activin A (100 ng/mL, #120-14; PeproTech, Rocky Hill, NJ, USA) from the next day for 3 days. BMP4 (50 ng/mL, #314-BP; R&D, Minneapolis, MN, USA) was added on the first day, and fetal bovine serum (FBS; #F7524; Sigma Aldrich, St. Louis, MO, USA) was included for the first 3 days at increasing concentrations of 0%, 0.2%, and 2.0%.

To generate foregut spheroids, RPMI-1640 medium supplemented with 2.0% FBS, 2 μM of CHIR99021 (#4423; TOCRIS, Bristol, UK), 500 ng/mL of FGF4 (#GFH31; Cell Guidance, St. Louis, MO, USA), and 200 ng/mL of Noggin (#6057-NG; R&D Systems) was added for 3 days until day 6. Retinoic acid (2 μM, #186-01114; WAKO) was added on day 6.

For the three-dimensional culture of gastric organoids using Matrigel (#354234; Corning, New York, NY, USA), spheroids were transferred to a three-dimensional *in vitro* culture system as previously described (McCracken et al., 2011). Spheroids were collected, mixed with 50 μL Matrigel, and plated into the middle of a well of a 4-well dish (#176740; Thermo Scientific). They were then cultured with advanced DMEM/F12 (#12634-010; Gibco) supplemented with N2 (1x) (#17502-001; Gibco), B27 (1x) (#17504-044; Gibco), 2 mM of L-glutamine (#25030-081; Gibco), 10 mM of HEPES (#15630-080; Gibco), penicillin/streptomycin, and 100 ng/mL of EGF (#236-EG; R&D). For the first 3 days of three-dimensional culture, 2 μM retinoic acid and 200 ng/mL Noggin were added to the media. The media was replaced every 2–4 days, as necessary. On day 20–21 (week 3), organoids were collected and re-plated with fresh Matrigel. Around days 41–43 (week 6), the organoids were transferred to fresh Matrigel again to prevent them from attaching to the bottom of the well of the plates. On day 56 (week 8), the organoids were analyzed by quantitative (q)RT-PCR, histological, immunohistological examination, and western blotting.

To inhibit HH and TGF-β signaling during MM differentiation of hGOs, cyclopamine (20 μM, #C9710; LKT Laboratories, St. Paul, MN, USA) and SB431542 (10 μM, #033-24631; Wako) were added to the medium, respectively, after re-plating the organoids on day 20–21.

### RNA isolation and PCR

Total RNA was extracted using TRIzol (#15596026; Life Technologies, Grand Island, NY). Human GOs were removed from Matrigel and homogenized using a beads crusher, μT-12 (#0068700-000; TAITECH, Saitama, Japan). Genomic DNA was removed by ezDNase (#11766051; Life Technologies), and reverse transcription was performed using a PrimeScript II 1st strand cDNA Synthesis Kit (#2690A; Takara Bio, Shiga, Japan) according to the manufacturer's protocol.

qRT-PCR was performed using TB Green *Premix Ex Taq* II (#RR820; Takara Bio) on a LightCycler 480 II (Roche, Basel, Switzerland). The PCR primers used for qRT-PCR were as follows: *GAPDH*, forward 5′-AGCCACATCGCTCAGACAC-3′, reverse 5′-GCCCAATACGACCAAATCC-3′; α*SMA*, forward 5′-TTCAATGTCCCAGCCATGTA-3′, reverse 5′-GAAGGAATAGCCACGCTCAG-3′; *MUC5AC*, forward 5′-CTCAGCTGTTCTCTGGACGA-3′, reverse 5′-GCTGGATGATCAGGCTCCTA-3′; *TFF-1*, forward 5′-TGTGCAAATAAGGGCTGCTG-3′, reverse 5′-GTCAGGATGCAGGCAGATCC-3′; *TFF-2*, forward 5′-AGTGCTGCTTCTCCAACTTC-3′, reverse 5′-AGATGCATCCTCTGGAACCAG-3′; *GLI1*, forward 5′-ACATCAACTCCGGCCAATAG-3′, reverse 5′-GAGGATGCTCCATTCTCTGG-3′; *GLI2*, forward 5′-AACACATGACCACCATGCAC-3′, reverse 5′-CTGCCACTGAAGTTTTCCAG-3′; *MYOCD*, forward 5′-TCAGCAGATGGATGAACTCCTG-3′, reverse 5′-AGTTGGACTTCGGGAAGATCTG-3′.

### RNA sequencing

The total RNA was isolated as described above and sent to Macrogen (Seoul, South Korea) for library preparation using the SMARTer stranded total RNA-seq kit. Paired-end RNA sequencing datasets were produced using an Illumina NovaSeq6000 (Illumina, San Diego, CA, USA). Reads were aligned to the human transcriptome (hg38) reference sequences using the Strand NGS software program (Strand Life Sciences, Bangalore, India). The heatmap was generated based on transcripts per million (TPM) values. Total RNA derived from two normal stomach samples purchased from BioChain Institute (R1234248-50; Newark, CA, USA) were also analyzed as controls. They were from two different donors. Although from which part of the stomach these samples were derived is unclear, based on the RNA-seq data, they were considered to have been taken from the antrum and at least deeper than the MM, as some adipose markers and an antral marker were expressed. Using the R software program, a PCA (in Figure 1E) was performed for the 8594 genes with TPM values exceeding 2 in all differentiated cells (weeks 1, 3, 6, and 8 and stomach). WikiPathways and Reactome were performed to identify pathways using the genes with a more than 10-fold change in their expression in the RNA-seq data according to the Strand NGS software program and R packages ReactomePA (Yu and He, 2016). A GSEA was performed using the GSEA software program (Subramanian et al., 2005).

### Histological and immunohistochemical examinations and in situ hybridization

The spheroids and organoids were fixed with 10% buffered neutral formalin solution for a period ranging from 3 h to overnight and embedded in paraffin blocks. They were sectioned at 4-μm thickness. After being deparaffinized and rehydrated, sections were stained with H&E. For immunostaining, antigens were retrieved by a pressure cooker with adequate retrieving buffer, and primary antibodies were incubated overnight at 4°C. The primary antibodies were as follows: anti-E-cadherin (CDH1) (goat polyclonal antibody, dilution 1:200; R&D AF648), anti-MUC5AC (mouse monoclonal antibody, CLH2, dilution 1:200; Abcam, Cambridge, MA, USA ab77576), and anti-αSMA (mouse monoclonal antibody, 1A4, dilution 1: 200; Dako, Carpinteria, CA, USA M0851), anti-Desmin (mouse monoclonal antibody, D33, dilution 1:200; Abcam ab8470), anti-SHH (rabbit monoclonal antibody, EP1190Y, dilution 1:200; Abcam ab53281), anti-TFF-1 (rabbit monoclonal antibody, EPR3972, dilution 1:200; Abcam ab92377), anti-TGF-β1 (rabbit polyclonal antibody, dilution 1:200; Abcam ab92486), anti-TGFβ Receptor I (rabbit polyclonal antibody, dilution 1:50; Abcam ab31013), anti-GLI1 (mouse monoclonal antibody, A-7, dilution 1:200, Santa Cruz, Dallas, TX, USA sc-515781), anti-IHH (rabbit polyclonal antibody, dilution 1:50; Abcam ab39634), and anti-HRH1 (rabbit polyclonal antibody, dilution 1:50; Proteintech 13413-1-AP).

For immunofluorescent staining, following the primary antibody, slides were washed in phosphate-buffered saline (PBS) and incubated with secondary antibodies (at dilution of 1:500) for 1 to 2 h at room temperature. Secondary antibodies were as follows: Donkey anti-goat IgG, Donkey anti-mouse IgG and Donkey anti-rabbit IgG (Alexa Fluor 488 and 594; Invitrogen, Carlsbad, CA, USA, A11055, A11058, A21202, A21203, A21206, A21207). Nuclei were stained with Hoechst 33342 (H3570; Thermo Scientific). Slides were washed in PBS, and coverslips were mounted using Fluoromount/Plus (K048; Diagnostic BioSystems, Pleasanton, CA, USA). The images were obtained with a fluorescence microscope (BZ-X700; Keyence, Osaka, Japan) and a confocal microscope (LSM700; Carl Zeiss, Jena, Germany).

For immunohistochemistry, following primary antibody incubation, incubation with a secondary antibody (Histofine Simple Stain MAX-PO; Nichirei Bioscience, Tokyo, Japan) was performed for 1 h at room temperature, and signals were detected using a Histofine DAB substrate kit (Nichirei). A normal stomach tissue array (BN01011b; US Biomax, Rockville, MD, USA) was stained with TGF-β1 as control.

For *in situ* hybridization, RNAscope technology (Advanced Cell Diagnostics, Newark, CA, USA) was used according to the manufacturer’s protocol. Slides were hybridized using the Hs_SHH (#600951; Advanced Cell Diagnostics) and the HS_TGFB1 probes (#400881; Advanced Cell Diagnostics). The signals were detected by RNAscope 2.5 HD Detection Kit RED (#322360; Advanced Cell Diagnostics). They were counterstained with hematoxylin or subjected to immunostaining with anti-CDH1 as a primary antibody and Donkey anti-goat IgG (Alexa Fluor 488) as a secondary antibody after appropriate antigen retrieval, as mentioned above.

### Human tissues

The IHC experiment for post-ESD gastric ulcer tissues was approved by the institutional review board of Kobe University Graduate School of Medicine and performed under protocol no. B200295. Eighteen post-ESD gastric ulcer cases were retrieved from the pathological archives of Kobe University Hospital between December 2017 and December 2019. All patients underwent gastric ESD for early gastric cancer, and gastrectomy was performed as an additional treatment within 3 months because of invasion at the deeper region of the submucosa, lymphovascular invasion, and poorly differentiated carcinoma components. Recurrence of gastric cancer was not found in any cases.

### Whole-mount staining

hGOs with and without attachment to the bottom of dishes were fixed with 4% paraformaldehyde (PFA) overnight at room temperature. Samples were permeabilized by PBS with 0.5% Triton X-(PBST) overnight at 4°C. Blocking was performed by PBST with 2% nonfat instant skim milk overnight at 4°C. Primary antibodies were diluted in PBST with 2% nonfat instant skim milk, and samples were incubated for 1 to 3 days at 4°C. Before incubating with secondary antibodies, samples were washed five times with PBST. Secondary antibodies were also diluted in PBST with 2% nonfat instant skim milk, and samples were incubated for 1 day at 4°C. An LSM700 (Carl Zeiss) was used for observation and image capture. Confocal images were acquired using a z stack collection with a 1-μm step. The images were processed for Z-projection (SD) using ImageJ/Fiji (Schindelin et al., 2012).

### Statistical analyses

All experiments were performed as at least three independent experiments. All data were analyzed using R (R Core Team, 2021). Differences in the mean values between two groups were analyzed using the paired or unpaired t test. Values of p < 0.05 were considered significant.

## Author contributions

K.U., T.K., M.K-A., T.I., and T.A. conceived the project and designed the experiments. K.U. and T.K. performed the culture experiments. K.U. performed immunostaining, western blotting, and qRT-PCR. K.U., M.K-A., and T.A. performed the bioinformatics analysis. T.A. supervised the project. K.U., M.K-A., and T.A. wrote the manuscript.

## Conflicts of interest

The authors declare no conflicts of interest in association with the present study.

## Data Availability

RNA-seq data have been deposited in the Gene Expression Omnibus (GEO) under accession number GSE174312, GSE178441, and GSE192526.

## Supplemental information

Document S1. Figures S1–S7 and Supplemental experimental procedures

Document S2. Article plus supplemental information
