# Supplementary material for: Epithelial-derived factors induce muscularis mucosa of human induced pluripotent stem cell-derived gastric organoids
Source: Stem Cell Reports. 2022 Mar 3;17(4):820–34. doi: 10.1016/j.stemcr.2022.02.002 (PMC9023774; doi:10.1016/j.stemcr.2022.02.002)
Supplement: Document S1. Figures S1–S7 and Supplemental experimental procedures [file mmc1.pdf]

Stem Cell Reports, Volume *17*

Supplemental Information

Epithelial-derived factors induce muscularis mucosa of human induced pluripotent stem cell-derived gastric organoids

Keiichiro Uehara, Michiyo Koyanagi-Aoi, Takahiro Koide, Tomoo Itoh, and Takashi Aoi

Fig. S1

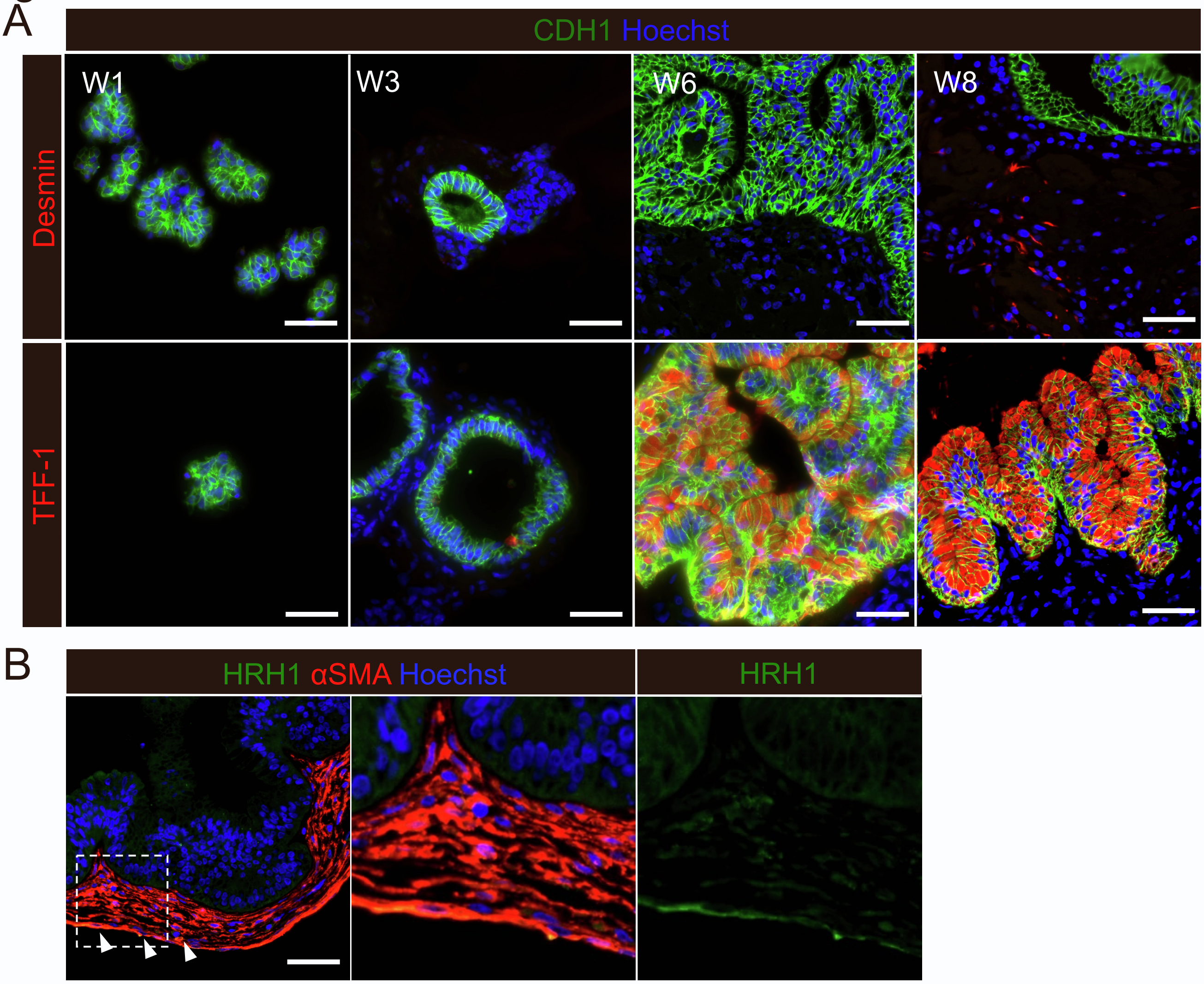

A
CDH1 Hoechst
Desmin
TFF-1
W1
W3
W6
W8
B
HRH1 αSMA Hoechst
HRH1

Figure S1. Induction of hGOs with MM derived from hiPSC. (A) IHC images of Desmin and TFF-1. Subepithelial cells were positive for Desmin at week 8. Epithelial cells were positive for TFF-1 at weeks 6 and 8. Scale bars, 50 µm. (B) IHC images of HRH1 and αSMA at week 8. Scale bars, 50 µm.

Fig. S2

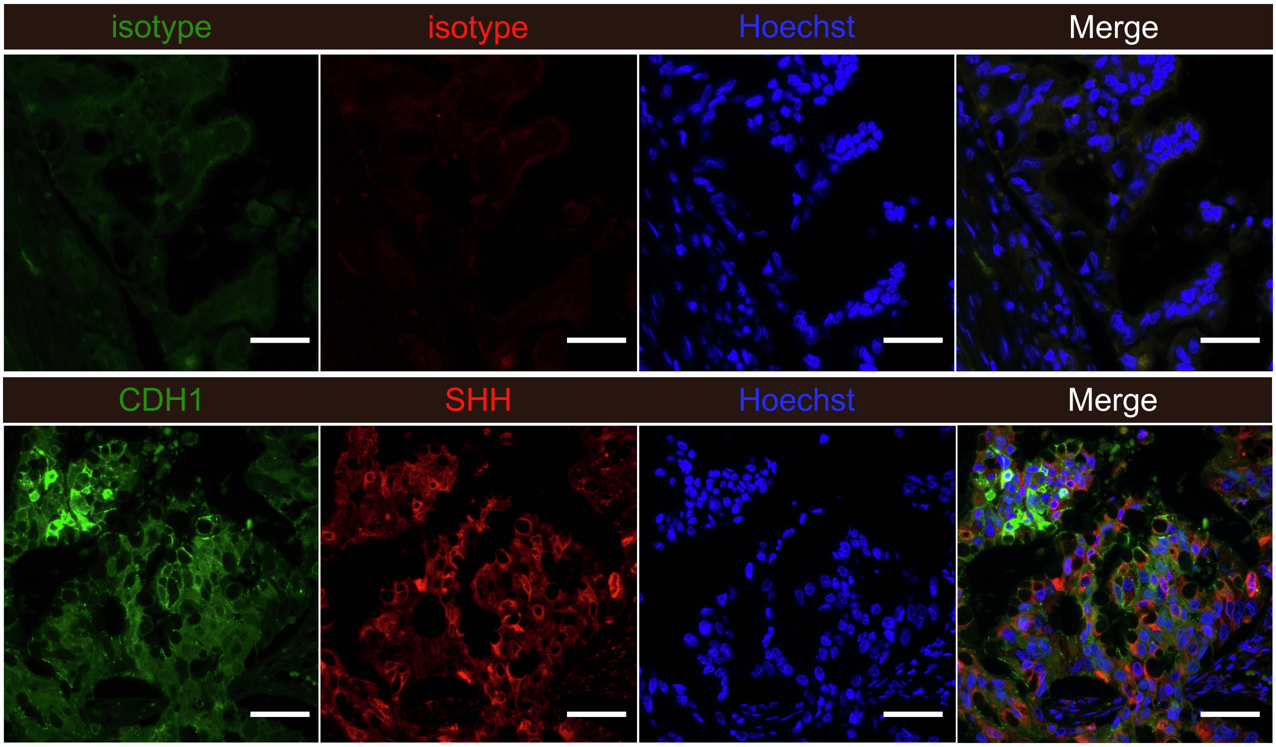

Figure S2. Negative and positive control for SHH IHC. Human pancreatic carcinoma was used as a control for SHH IHC. Scale bars, 50 μm.

Fig. S3

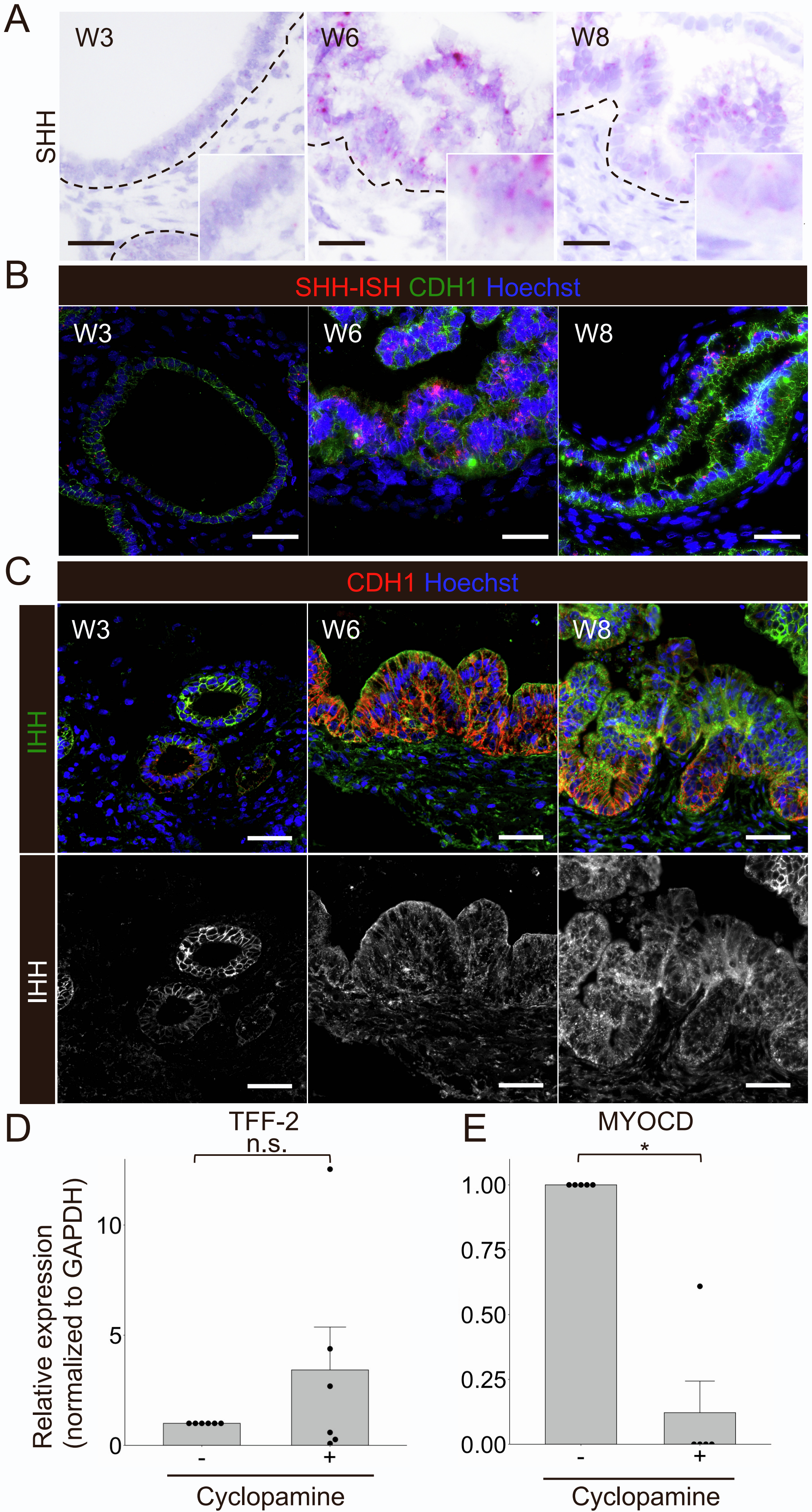
A
SHH
W3
W6
W8
B
SHH-ISH CDH1 Hoechst
W3
W6
W8
C
CDH1 Hoechst
IHH
IHH
W3
W6
W8
D
TFF-2
n.s.
Relative expression
(normalized to GAPDH)
0
5
10
-
+
Cyclopamine
E
MYOCD
*
0.00
0.25
0.50
0.75
1.00
-
+
Cyclopamine

Figure S3. Hedgehog signaling of hGOs. (A) Light microscope images showed SHH mRNA (red). Dotted lines indicate the borders between the epithelium and subepithelium. Scale bars, 50 μm. (B) Fluorescent microscope images showed SHH mRNA (red) and CDH1 (green). Scale bars, 50 μm. (C) Immunohistochemistry for IHH. Scale bars, 50 μm. (D)(E) Results of a qRT-PCR analysis of TFF-2 (D) and MYOCD (E) of hGOs with or without cyclopamine. Normalized to control. TFF-2, number of independent differentiation experiments: n=6; MYOCD, n=5. Mean values ± SE, unpaired *t* test: *$p<0.01$.

Fig. S4

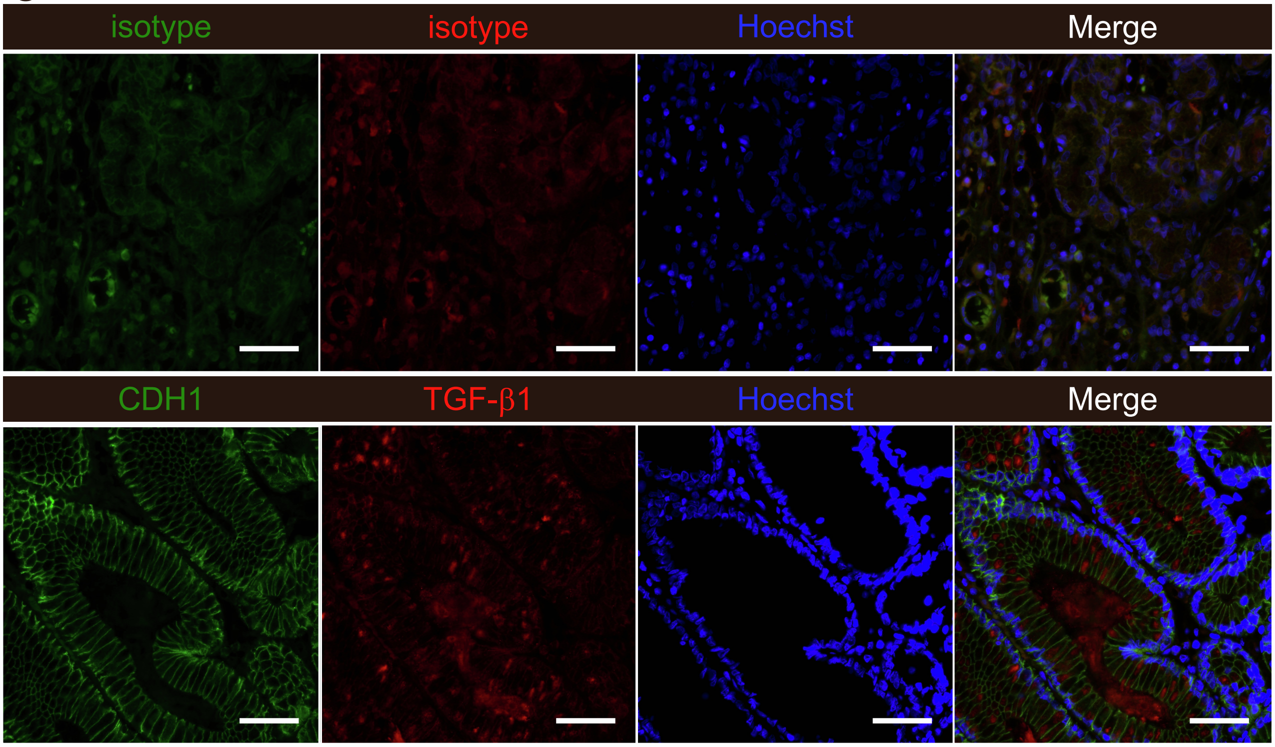

Figure S4. Negative and positive control for TGF-β1 IHC. Human stomach was used as a control for TGF-β1 IHC. Autofluorescence by red blood cells was observed in negative control figures. Scale bars, 50 μm.

Fig. S5

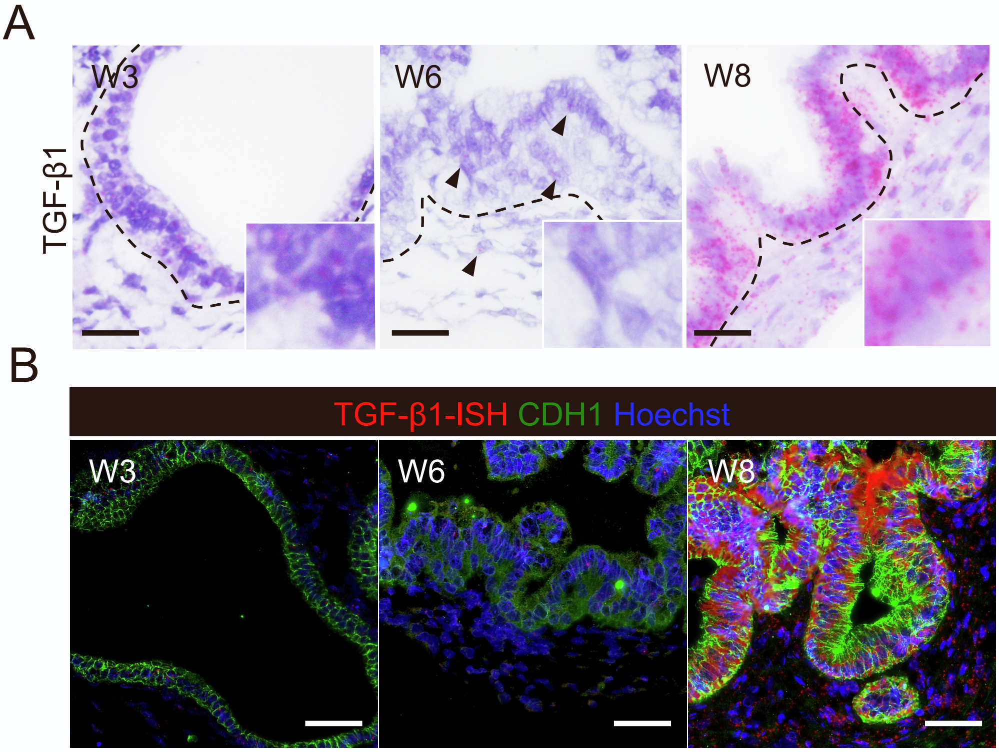

Figure S5. TGF-β1 *in situ* hybridization of hGOs. (A) Light microscope images showed TGF-β1 mRNA (red). Dotted lines indicate borders between epithelium and subepithelium. Scale bars, 50 μm. (B) Fluorescent microscope images showed TGF-β1 mRNA (red) and CDH1 (green). Scale bars, 50 μm.

Fig. S6

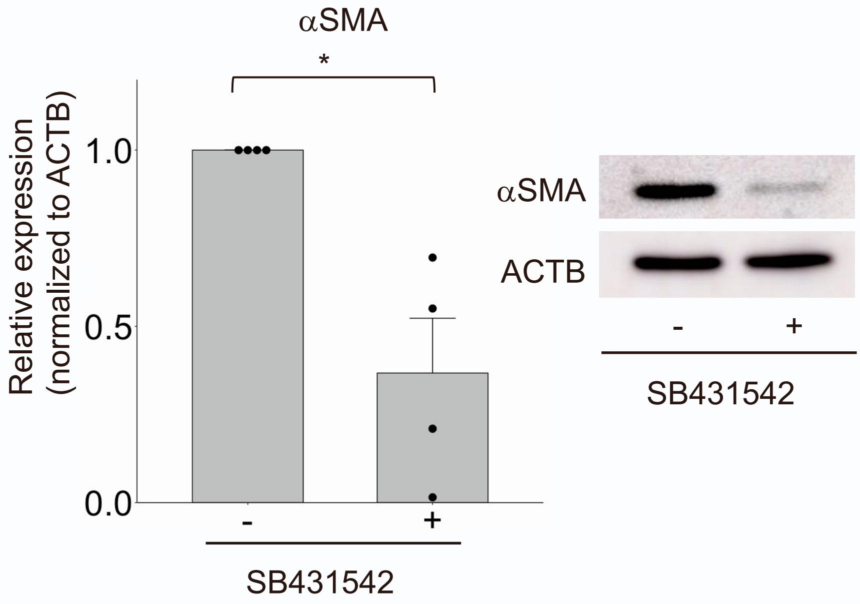
αSMA
*
Relative expression
(normalized to ACTB)
1.0
0.5
0.0
-
+
SB431542
αSMA
ACTB
-
+
SB431542

Figure S6. SB431542 reduced MM of hGOs. The expression of αSMA in hGOs with or without SB431542 was examined by western blotting. Normalized to control. n=4 independent experiments. Mean values ± SE, unpaired *t* test: *p<0.01.

Fig. S7

A

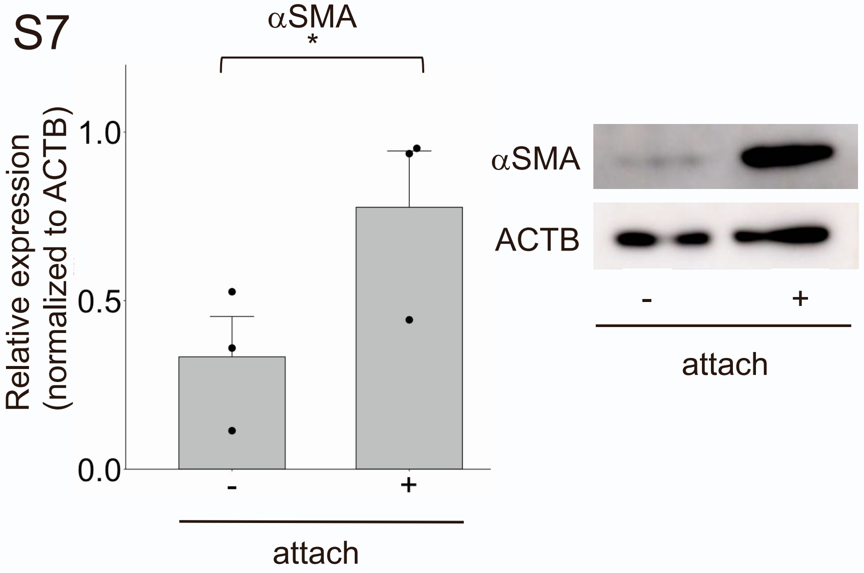
αSMA
*
Relative expression
(normalized to ACTB)
1.0
0.5
0.0
-
+
attach
αSMA
ACTB
-
+
attach

B

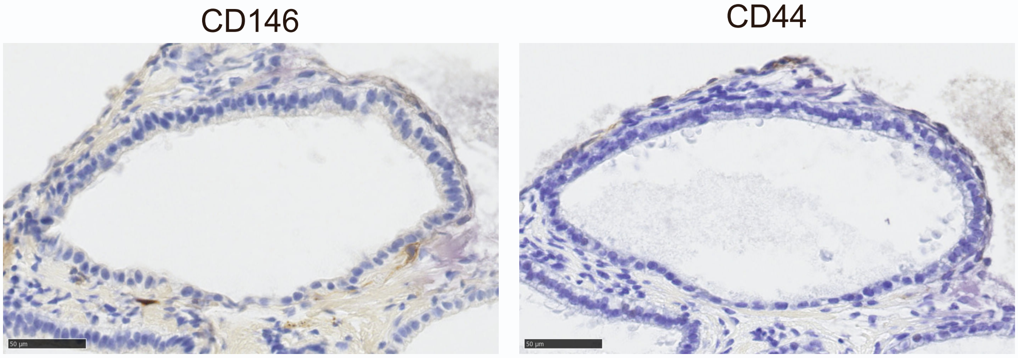
CD146
CD44

Figure S7. The mechanical environment promotes the subepithelial αSMA expression. (A) The expression of αSMA in hGOs with or without attachment to the surface of the dishes was examined by western blotting. n=3 independent experiments. Mean values ± SE, paired *t* test: *$p<0.05$. (B) IHC images of CD146 and CD44. Positive spindle cells were found around hGOs at week 3. Scale bars, 50 μm.

Supplemental experimental procedures

Western blot analyses

hGOs were removed from Matrigel and lysed in M-PER Mammalian Protein Extraction Reagent (78501; Life Technologies) supplemented with cOmplete (11697498001; Roche). The lysates were subjected to sodium dodecyl sulfate-polyacrylamide gel electrophoresis (SDS-PAGE). The gels were transferred to polyvinylidene fluoride membranes (IPVH00010; Merck, Frankfurt, Germany) using iBlot (Life Technologies), and immunoblotting was performed using iBind (Life Technologies). Primary antibodies were anti-β-actin (A5441; mouse monoclonal antibody, AC-15, dilution 1:3000; Sigma-Aldrich) and anti-αSMA (mouse monoclonal antibody, 1A4, dilution 1:1000; Dako). The secondary antibody was anti-mouse IgG (7076S; HRP-linked antibody, dilution 1:3000; Cell Signaling Technology, Danvers, MA, USA). The images were obtained using the Amersham Imager 600 (GE Healthcare, Chicago, IL, USA).
